# 2,3-Bis{[2,3-dimethyl-6-(phenyl­vin­yl)phen­yl]imino}­butane

**DOI:** 10.1107/S1600536814000440

**Published:** 2014-01-11

**Authors:** Jie Zhao, Jianchao Yuan, Weibing Xu, Jingjing Chen, Yanqiong Mu

**Affiliations:** a Key Laboratory of Eco-Environment-Related Polymer Materials of the Ministry of Education, Key Laboratory of Polymer Materials of Gansu Province, College of Chemistry & Chemical Engineering, Northwest Normal University, Lanzhou 730070, People’s Republic of China

## Abstract

In the title compound, C_36_H_36_N_2_, a product of the condensation reaction of 2,3-dimethyl-6-phenyl­vinyl­benzenamine and 2,3-butane­dione, the complete mol­ecule is generated by the application of an inversion centre. The central C—C bond in the 1,4-di­aza­butadiene fragment is *trans*-configured and situated about the inversion center. The dihedral angle between the ring attached to N and the 1,4-di­aza­butadiene plane is 78.24 (36)°, while the 1,4-di­aza­butadiene plane makes an angle of 30.71 (26)° with the phenyl ring.

## Related literature   

The title compound was synthesized as an α-di­imine ligand for transtion metals, see: Johnson *et al.* (1995 ▶); Gao *et al.* (2012 ▶); Zhang & Ye (2012 ▶); Sun *et al.* (2012 ▶); Popeney *et al.* (2012 ▶); Shi *et al.* (2012 ▶). For related structures, see: Helldörfer, Milius & Alt (2003 ▶); Helldörfer, Backhaus & Alt (2003 ▶); Popeney & Guan (2005 ▶); Kravchenko & Waymouth (1998 ▶).
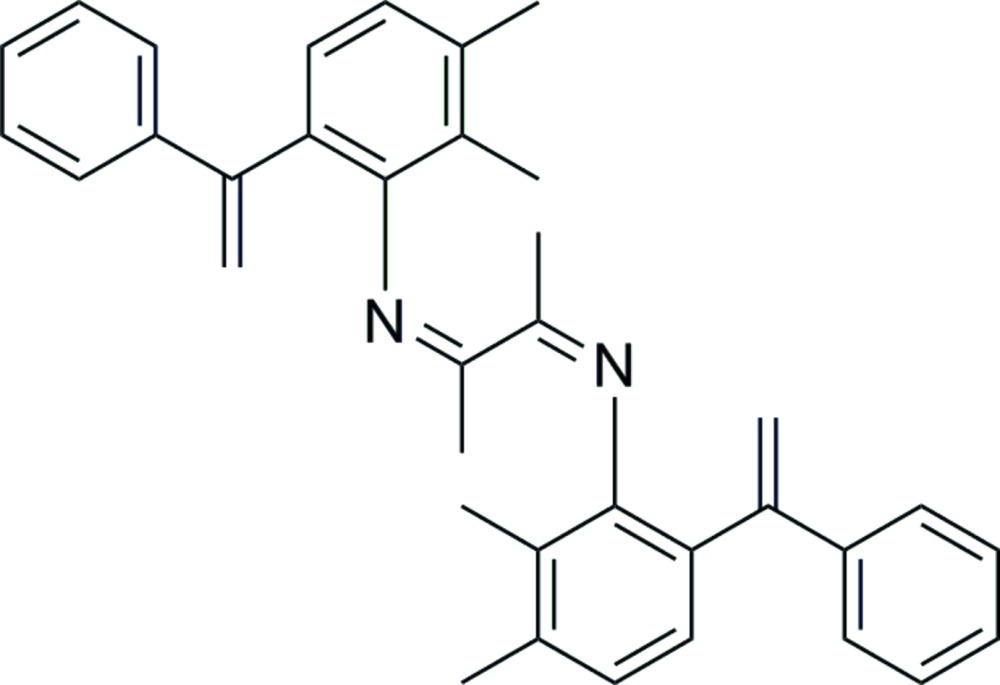


## Experimental   

### 

#### Crystal data  

C_36_H_36_N_2_
*M* *_r_* = 496.67
Monoclinic, 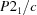

*a* = 9.613 (8) Å
*b* = 16.285 (14) Å
*c* = 9.639 (8) Å
β = 101.679 (9)°
*V* = 1478 (2) Å^3^
*Z* = 2
Mo *K*α radiation
μ = 0.06 mm^−1^
*T* = 296 K
0.26 × 0.24 × 0.18 mm

#### Data collection  

Bruker APEXII CCD diffractometer
Absorption correction: multi-scan (*SADABS*; Bruker, 2008 ▶) *T*_min_ = 0.984, *T*_max_ = 0.989
10305 measured reflections
2693 independent reflections
1374 reflections with *I* > 2σ(*I*)
*R*_int_ = 0.041

#### Refinement  

*R*[*F* ^2^ > 2σ(*F* ^2^)] = 0.103
*wR*(*F* ^2^) = 0.327
*S* = 1.03
2693 reflections
175 parameters
84 restraints
H-atom parameters constrained
Δρ_max_ = 0.46 e Å^−3^
Δρ_min_ = −0.42 e Å^−3^

### 

Data collection: *APEX2* (Bruker, 2008 ▶); cell refinement: *SAINT* (Bruker, 2008 ▶); data reduction: *SAINT*; program(s) used to solve structure: *SHELXS97* (Sheldrick, 2008 ▶); program(s) used to refine structure: *SHELXL97* (Sheldrick, 2008 ▶); molecular graphics: *SHELXTL*; software used to prepare material for publication: *SHELXTL*.

## Supplementary Material

Crystal structure: contains datablock(s) I, New_Global_Publ_Block. DOI: 10.1107/S1600536814000440/qm2103sup1.cif

Structure factors: contains datablock(s) I. DOI: 10.1107/S1600536814000440/qm2103Isup2.hkl

Supporting information file. DOI: 10.1107/S1600536814000440/qm2103Isup3.cml

CCDC reference:

Additional supporting information:  crystallographic information; 3D view; checkCIF report

## Figures and Tables

**Fig. 1.**
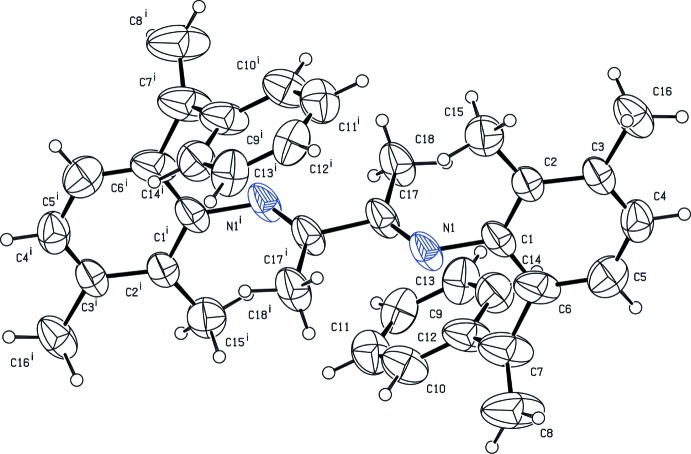
The title molecule with displacement ellipsoids plotted at 30% probability level. Primed atoms are related by the symmetry code (-x + 1, -y + 2, -z + 1).

**Fig. 2.**
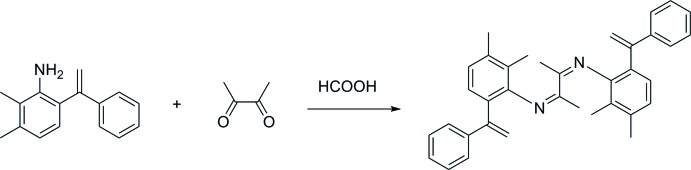
A condensation reaction of 2,3-butanedione and 2,3-dimethyl-6-phenylvinylbenzenamine.

## supplementary crystallographic information

### 1. Comment

The discovery of Ni(II) and Pd(II) α-olefin polymerization catalysts containing a bulky α-diimine ligand, [*MX*_2_(α-diimine)](*M*=Ni,Pd;*X*=halide), by Brookhart and co-workers has stimulated renewed interest in the chemistry of 1,4-diazadiene ligands and their complexes (Johnson *et al.*, 1995; Gao *et al.*, 2012; Zhang *et al.*, 2012; Sun *et al.*, 2012; Popeney *et al.*, 2012; Shi *et al.*, 2012). The catalyst activity and properties of the resulting polymers are greatly dependent on the reaction conditions (Helldörfer *et al.*, 2003) and ligand structure (Popeney *et al.*, 2005; Helldörfer *et al.*, 2003; Kravchenko & Waymouth, 1998). In this study, we designed and synthesized the title compound as a abidentate ligand (Fig. 1). The complete molecule is generated by the application of an inversion centre. The central C—C bond in the 1,4-diazabutadiene fragment is *trans*-configured and situated on an inversion center as shown in Fig. 1. Neither hydrogen bonding nor aromatic stacking are observed in the crystal structure.

### 2. Experimental

Formic acid (0.5 ml) was added to a stirred solution of 2,3-butanedione (0.09 g, 1.00 mmol) and 2,3-dimethyl-6-phenylvinylbenzenamine (0.49 g, 2.2 mmol) in ethanol (10 ml) (Fig. 2). The mixture was refluxed for 24 h, and then cooled and the precipitate was separated by filtration. The solid was recrystallized from EtOH/CH_2_Cl_2_ (*v*/*v*= 10:1), washed and dried under vacuum. Yield: 0.38 g (76%). Crystals suitable for *X*–raystructure determination were grown from a acyclohexane/dichloromethane (v:v= 1:2) solution. Anal. Calc. for C_36_H_36_N_2_: C, 86.35; H, 8.05; N, 5.59.Found: C,88.39; H, 8.09; N, 5.42.

### 3. Refinement

All hydrogen atoms were placed in calculated positions with C—H distances of 0.93 Å and 0.96 Å for aryl and methyl H atoms. They were included in the refinement in a riding model approximation, respectively. The H atoms were assigned *U*_iso_ = 1.2Ueq(C) for aryl H and *U*_iso_ = 1.5Ueq(C) for methyl H.

### Crystal data

**Table:**

C_36_H_36_N_2_	*F*(000) = 532
*M**_r_* = 496.67	*D*_x_ = 1.116 Mg m^−^^3^
Monoclinic, *P*2_1_/*c*	Mo *K*α radiation, λ = 0.71073 Å
*a* = 9.613 (8) Å	Cell parameters from 1803 reflections
*b* = 16.285 (14) Å	θ = 2.5–22.5°
*c* = 9.639 (8) Å	µ = 0.06 mm^−^^1^
β = 101.679 (9)°	*T* = 296 K
*V* = 1478 (2) Å^3^	Block, yellow
*Z* = 2	0.26 × 0.24 × 0.18 mm

### Data collection

**Table:**

Bruker APEXII CCD diffractometer	2693 independent reflections
Radiation source: fine-focus sealed tube	1374 reflections with *I* > 2σ(*I*)
Graphite monochromator	*R*_int_ = 0.041
φ and ω scans	θ_max_ = 25.3°, θ_min_ = 2.2°
Absorption correction: multi-scan (*SADABS*; Bruker, 2008)	*h* = −11→11
*T*_min_ = 0.984, *T*_max_ = 0.989	*k* = −19→19
10305 measured reflections	*l* = −11→10

### Refinement

**Table:**

Refinement on *F*^2^	Primary atom site location: structure-invariant direct methods
Least-squares matrix: full	Secondary atom site location: difference Fourier map
*R*[*F*^2^ > 2σ(*F*^2^)] = 0.103	Hydrogen site location: inferred from neighbouring sites
*wR*(*F*^2^) = 0.327	H-atom parameters constrained
*S* = 1.03	*w* = 1/[σ^2^(*F*_o_^2^) + (0.1098*P*)^2^ + 2.2955*P*] where *P* = (*F*_o_^2^ + 2*F*_c_^2^)/3
2693 reflections	(Δ/σ)_max_ < 0.001
175 parameters	Δρ_max_ = 0.46 e Å^−^^3^
84 restraints	Δρ_min_ = −0.42 e Å^−^^3^

### Special details

**Table:**

Geometry. All e.s.d.'s (except the e.s.d. in the dihedral angle between two l.s. planes) are estimated using the full covariance matrix. The cell e.s.d.'s are taken into account individually in the estimation of e.s.d.'s in distances, angles and torsion angles; correlations between e.s.d.'s in cell parameters are only used when they are defined by crystal symmetry. An approximate (isotropic) treatment of cell e.s.d.'s is used for estimating e.s.d.'s involving l.s. planes.
Refinement. Refinement of *F*^2^ against ALL reflections. The weighted *R*-factor *wR* and goodness of fit *S* are based on *F*^2^, conventional *R*-factors *R* are based on *F*, with *F* set to zero for negative *F*^2^. The threshold expression of *F*^2^ > σ(*F*^2^) is used only for calculating *R*-factors(gt) *etc*. and is not relevant to the choice of reflections for refinement. *R*-factors based on *F*^2^ are statistically about twice as large as those based on *F*, and *R*- factors based on ALL data will be even larger.

### Fractional atomic coordinates and isotropic or equivalent isotropic displacement parameters (Å^2^)

**Table:**

	*x*	*y*	*z*	*U*_iso_*/*U*_eq_	
C1	0.7542 (5)	0.9715 (4)	0.3661 (7)	0.0873 (16)	
C2	0.8434 (5)	1.0392 (4)	0.3706 (5)	0.0738 (15)	
C3	0.9406 (5)	1.0398 (4)	0.2810 (6)	0.0795 (16)	
C4	0.9452 (6)	0.9752 (5)	0.1928 (7)	0.0954 (19)	
H4	1.0072	0.9770	0.1303	0.114*	
C5	0.8604 (7)	0.9073 (5)	0.1938 (8)	0.107 (2)	
H5	0.8682	0.8633	0.1344	0.128*	
C6	0.7627 (6)	0.9037 (4)	0.2827 (9)	0.112 (2)	
C7	0.6752 (7)	0.8283 (4)	0.2878 (10)	0.135 (2)	
C8	0.7248 (9)	0.7605 (5)	0.3422 (13)	0.175 (3)	
H8A	0.8211	0.7558	0.3819	0.210*	
H8B	0.6649	0.7157	0.3421	0.210*	
C9	0.5196 (6)	0.8376 (4)	0.2159 (9)	0.116 (2)	
C10	0.4124 (7)	0.8072 (4)	0.2744 (8)	0.108 (2)	
H10	0.4340	0.7810	0.3619	0.130*	
C11	0.2722 (7)	0.8147 (5)	0.2058 (9)	0.110 (2)	
H11	0.2004	0.7944	0.2481	0.132*	
C12	0.2387 (7)	0.8512 (4)	0.0777 (8)	0.101 (2)	
H12	0.1442	0.8555	0.0313	0.121*	
C13	0.3433 (7)	0.8816 (5)	0.0169 (7)	0.109 (2)	
H13	0.3207	0.9069	−0.0713	0.131*	
C14	0.4836 (7)	0.8750 (4)	0.0858 (8)	0.107 (2)	
H14	0.5547	0.8961	0.0436	0.128*	
C15	0.8381 (7)	1.1086 (4)	0.4719 (7)	0.107 (2)	
H15A	0.7756	1.1506	0.4254	0.160*	
H15B	0.9317	1.1309	0.5026	0.160*	
H15C	0.8038	1.0886	0.5524	0.160*	
C16	1.0396 (6)	1.1122 (4)	0.2799 (7)	0.109 (2)	
H16A	1.0944	1.1040	0.2081	0.163*	
H16B	1.1023	1.1168	0.3707	0.163*	
H16C	0.9848	1.1617	0.2601	0.163*	
C17	0.5439 (5)	1.0046 (4)	0.4443 (6)	0.0981 (17)	
C18	0.4816 (6)	1.0561 (5)	0.3171 (7)	0.114 (2)	
H18A	0.5390	1.0508	0.2468	0.171*	
H18B	0.4794	1.1126	0.3450	0.171*	
H18C	0.3868	1.0378	0.2783	0.171*	
N1	0.6610 (4)	0.9671 (3)	0.4649 (5)	0.0950 (14)	

### Atomic displacement parameters (Å^2^)

**Table:**

	*U*^11^	*U*^22^	*U*^33^	*U*^12^	*U*^13^	*U*^23^
C1	0.049 (2)	0.109 (4)	0.104 (4)	0.019 (3)	0.016 (2)	0.067 (3)
C2	0.052 (3)	0.095 (4)	0.072 (3)	0.016 (3)	0.008 (2)	0.035 (3)
C3	0.051 (3)	0.109 (4)	0.077 (4)	0.006 (3)	0.010 (3)	0.040 (3)
C4	0.070 (4)	0.131 (6)	0.089 (4)	0.020 (4)	0.023 (3)	0.024 (4)
C5	0.077 (4)	0.118 (6)	0.120 (5)	0.021 (4)	0.005 (4)	0.006 (4)
C6	0.058 (3)	0.089 (4)	0.177 (5)	0.011 (3)	−0.004 (3)	0.057 (4)
C7	0.081 (3)	0.098 (3)	0.207 (5)	0.002 (3)	−0.021 (3)	0.063 (3)
C8	0.105 (5)	0.098 (5)	0.294 (9)	0.008 (4)	−0.026 (6)	0.051 (6)
C9	0.070 (3)	0.085 (4)	0.180 (5)	−0.001 (3)	−0.007 (4)	0.047 (4)
C10	0.103 (5)	0.104 (5)	0.107 (5)	−0.026 (4)	−0.005 (4)	0.017 (4)
C11	0.092 (5)	0.123 (6)	0.115 (6)	−0.036 (4)	0.022 (4)	−0.024 (5)
C12	0.081 (4)	0.129 (6)	0.089 (5)	0.007 (4)	0.007 (4)	−0.030 (4)
C13	0.088 (4)	0.150 (6)	0.085 (4)	0.021 (4)	0.011 (4)	0.004 (4)
C14	0.080 (4)	0.119 (5)	0.122 (5)	0.017 (4)	0.021 (4)	0.030 (4)
C15	0.100 (5)	0.119 (5)	0.103 (5)	0.017 (4)	0.023 (4)	0.025 (4)
C16	0.067 (3)	0.142 (6)	0.115 (5)	−0.011 (4)	0.010 (3)	0.050 (4)
C17	0.058 (2)	0.133 (4)	0.107 (3)	0.022 (3)	0.026 (2)	0.076 (3)
C18	0.081 (4)	0.159 (5)	0.108 (4)	0.036 (4)	0.030 (3)	0.082 (4)
N1	0.054 (2)	0.127 (3)	0.107 (3)	0.020 (2)	0.024 (2)	0.073 (2)

### Geometric parameters (Å, º)

**Table:**

C1—C6	1.379 (10)	C11—C12	1.350 (9)
C1—C2	1.392 (8)	C11—H11	0.9300
C1—N1	1.435 (7)	C12—C13	1.356 (9)
C2—C3	1.395 (7)	C12—H12	0.9300
C2—C15	1.500 (8)	C13—C14	1.381 (9)
C3—C4	1.359 (9)	C13—H13	0.9300
C3—C16	1.516 (8)	C14—H14	0.9300
C4—C5	1.375 (9)	C15—H15A	0.9600
C4—H4	0.9300	C15—H15B	0.9600
C5—C6	1.395 (9)	C15—H15C	0.9600
C5—H5	0.9300	C16—H16A	0.9600
C6—C7	1.495 (9)	C16—H16B	0.9600
C7—C8	1.273 (9)	C16—H16C	0.9600
C7—C9	1.523 (9)	C17—N1	1.261 (6)
C8—H8A	0.9300	C17—C17^i^	1.502 (10)
C8—H8B	0.9300	C17—C18	1.506 (7)
C9—C10	1.364 (9)	C18—H18A	0.9600
C9—C14	1.373 (9)	C18—H18B	0.9600
C10—C11	1.381 (9)	C18—H18C	0.9600
C10—H10	0.9300		
			
C6—C1—C2	123.0 (5)	C10—C11—H11	119.8
C6—C1—N1	117.7 (6)	C11—C12—C13	119.7 (6)
C2—C1—N1	118.8 (7)	C11—C12—H12	120.1
C1—C2—C3	118.2 (6)	C13—C12—H12	120.1
C1—C2—C15	121.0 (5)	C12—C13—C14	120.0 (7)
C3—C2—C15	120.8 (6)	C12—C13—H13	120.0
C4—C3—C2	119.5 (6)	C14—C13—H13	120.0
C4—C3—C16	119.8 (6)	C9—C14—C13	121.0 (6)
C2—C3—C16	120.7 (6)	C9—C14—H14	119.5
C3—C4—C5	121.6 (6)	C13—C14—H14	119.5
C3—C4—H4	119.2	C2—C15—H15A	109.5
C5—C4—H4	119.2	C2—C15—H15B	109.5
C4—C5—C6	120.8 (7)	H15A—C15—H15B	109.5
C4—C5—H5	119.6	C2—C15—H15C	109.5
C6—C5—H5	119.6	H15A—C15—H15C	109.5
C1—C6—C5	116.8 (6)	H15B—C15—H15C	109.5
C1—C6—C7	122.6 (8)	C3—C16—H16A	109.5
C5—C6—C7	120.5 (9)	C3—C16—H16B	109.5
C8—C7—C6	124.0 (6)	H16A—C16—H16B	109.5
C8—C7—C9	121.7 (7)	C3—C16—H16C	109.5
C6—C7—C9	114.3 (5)	H16A—C16—H16C	109.5
C7—C8—H8A	120.0	H16B—C16—H16C	109.5
C7—C8—H8B	120.0	N1—C17—C17^i^	116.8 (5)
H8A—C8—H8B	120.0	N1—C17—C18	126.4 (5)
C10—C9—C14	117.8 (6)	C17^i^—C17—C18	116.7 (5)
C10—C9—C7	122.0 (7)	C17—C18—H18A	109.5
C14—C9—C7	120.1 (7)	C17—C18—H18B	109.5
C9—C10—C11	121.0 (7)	H18A—C18—H18B	109.5
C9—C10—H10	119.5	C17—C18—H18C	109.5
C11—C10—H10	119.5	H18A—C18—H18C	109.5
C12—C11—C10	120.4 (7)	H18B—C18—H18C	109.5
C12—C11—H11	119.8	C17—N1—C1	121.8 (4)
			
C6—C1—C2—C3	3.4 (7)	C1—C6—C7—C9	74.5 (10)
N1—C1—C2—C3	175.4 (4)	C5—C6—C7—C9	−106.9 (8)
C6—C1—C2—C15	−174.9 (5)	C8—C7—C9—C10	44.8 (14)
N1—C1—C2—C15	−2.9 (7)	C6—C7—C9—C10	−137.7 (8)
C1—C2—C3—C4	0.1 (7)	C8—C7—C9—C14	−132.8 (10)
C15—C2—C3—C4	178.4 (5)	C6—C7—C9—C14	44.7 (12)
C1—C2—C3—C16	179.4 (5)	C14—C9—C10—C11	−0.8 (11)
C15—C2—C3—C16	−2.4 (7)	C7—C9—C10—C11	−178.5 (7)
C2—C3—C4—C5	−2.9 (8)	C9—C10—C11—C12	1.1 (11)
C16—C3—C4—C5	177.9 (5)	C10—C11—C12—C13	−0.8 (11)
C3—C4—C5—C6	2.2 (9)	C11—C12—C13—C14	0.1 (11)
C2—C1—C6—C5	−4.1 (8)	C10—C9—C14—C13	0.1 (11)
N1—C1—C6—C5	−176.1 (5)	C7—C9—C14—C13	177.9 (7)
C2—C1—C6—C7	174.6 (5)	C12—C13—C14—C9	0.2 (11)
N1—C1—C6—C7	2.5 (8)	C17^i^—C17—N1—C1	−179.8 (7)
C4—C5—C6—C1	1.3 (9)	C18—C17—N1—C1	1.9 (12)
C4—C5—C6—C7	−177.4 (6)	C6—C1—N1—C17	−106.0 (7)
C1—C6—C7—C8	−108.0 (11)	C2—C1—N1—C17	81.6 (8)
C5—C6—C7—C8	70.6 (13)		

Symmetry code: (i) −*x*+1, −*y*+2, −*z*+1.
